# Oxygen Tension Modulates Differentiation and Primary Macrophage Functions in the Human Monocytic THP-1 Cell Line

**DOI:** 10.1371/journal.pone.0054926

**Published:** 2013-01-23

**Authors:** Ana Cristina G. Grodzki, Cecilia Giulivi, Pamela J. Lein

**Affiliations:** Department of Molecular Biosciences, School of Veterinary Medicine, University of California Davis, Davis, California, United States of America; University of North Dakota, United States of America

## Abstract

The human THP-1 cell line is widely used as an *in vitro* model system for studying macrophage differentiation and function. Conventional culture conditions for these cells consist of ambient oxygen pressure (∼20% v/v) and medium supplemented with the thiol 2-mercaptoethanol (2-ME) and serum. In consideration of the redox activities of O_2_ and 2-ME, and the extensive experimental evidence supporting a role for reactive oxygen species (ROS) in the differentiation and function of macrophages, we addressed the question of whether culturing THP-1 cells under a more physiologically relevant oxygen tension (5% O_2_) in the absence of 2-ME and serum would alter THP-1 cell physiology. Comparisons of cultures maintained in 18% O_2_ *versus* 5% O_2_ indicated that reducing oxygen tension had no effect on the proliferation of undifferentiated THP-1 cells. However, decreasing the oxygen tension to 5% O_2_ significantly increased the rate of phorbol ester-induced differentiation of THP-1 cells into macrophage-like cells as well as the metabolic activity of both undifferentiated and PMA-differentiated THP-1 cells. Removal of both 2-ME and serum from the medium decreased the proliferation of undifferentiated THP-1 cells but increased metabolic activity and the rate of differentiation under either oxygen tension. In differentiated THP-1 cells, lowering the oxygen tension to 5% O_2_ decreased phagocytic activity, the constitutive release of β-hexosaminidase and LPS-induced NF-κB activation but enhanced LPS-stimulated release of cytokines. Collectively, these data demonstrate that oxygen tension influences THP-1 cell differentiation and primary macrophage functions, and suggest that culturing these cells under tightly regulated oxygen tension in the absence of exogenous reducing agent and serum is likely to provide a physiologically relevant baseline from which to study the role of the local redox environment in regulating THP-1 cell physiology.

## Introduction

While it is widely accepted that immortalized cell lines do not exactly replicate primary human cells, cell lines can be extremely powerful experimental models and are generally more widely accessible to the research community than primary human cells. However, there is increasing awareness that cell culture conditions can significantly influence cellular differentiation and function *in vitro*, and thus, it is extremely important to determine whether changing specific cell culture parameters influences the fidelity by which cell lines replicate the functions performed by primary cell types. The THP-1 cell line was originally derived from human monocytes approximately 30 years ago [1], and has becoming a widely used *in vitro* model system for studying the differentiation, physiology and pharmacology of monocytes and macrophages. Like most commonly used cell lines, THP-1 cells are typically maintained in culture at atmospheric oxygen tension ((18–21% O_2_ v/v) in medium supplemented with the reducing agent 2-mercaptoethanol (2-ME) and serum. While cells in certain microenvironments, such as the alveoli of the mammalian lung, may encounter oxygen tensions approaching atmospheric levels, normoxic levels in most mammalian tissues range from 3 to 12% O_2_ (v/v) [2]. Hyperoxia increases intracellular levels of reactive oxygen species (ROS) [3] and, thus, conventional culture conditions may predispose cells to oxidative stress. The supplementation of culture medium with 2-ME and serum likely provides some protection against the oxidative stress generated in cells cultured under atmospheric oxygen tension. Maintaining intracellular reserves of reduced glutathione (GSH) is critical to maintaining intracellular redox homeostasis [4], and as a reducing agent, 2-ME can facilitate the maintenance of reduced levels of thiol-containing proteins and peptides. 2-ME was originally added to media used to culture murine lymphocytes to increase intracellular levels of reduced glutathione and thereby enhance cellular functions [5]; however, ME does not enter the cells freely but does increase uptake of Cys which may result in increased GSH synthesis. This practice has since been adopted and recommended for culturing diverse cell types derived from multiple species, including human THP-1 cells, with little experimental evidence to support its value in enhancing cell viability and/or cell-specific functions.

Given the influence of ambient oxygen tension on redox reactions, and the thiol-reducing activity of 2-ME, it seems likely that changing these culture parameters will influence the redox balance in the cell. This in turn is likely to have significant impacts on cellular functions since intracellular ROS levels are tightly regulated not only to prevent oxidative stress-induced cell damage, but also because ROS are crucial signaling molecules in energy production, phagocytosis [6], and cellular differentiation [7]. Moreover, there is evidence that some of the same transcription factors that are activated by oxidative stress, such as NF-κB and AP-1, are also involved in mediating the effects of ROS on other cellular functions, such as cytokine production [8]. Consistent with the proposed role of ROS in normal cell physiology, changes in oxygen tension have been shown to modulate cell proliferation [9], maturation [10], differentiation [2] and cytokine production [11]–[13]. For example, studies have demonstrated that the exceptionally low oxygen tensions associated with the tumor environment are causally linked to upregulation of transcription factors that enhance cytokine production in tumor-associated macrophages [14].

The goal of this study was to determine whether culture conditions, specifically reducing agents and oxygen tension, have a significant influence on the macrophage functions of THP-1 cells. The answer to this question has important implications with respect to optimizing THP-1 cell culture to better replicate primary human macrophages, and for interpreting results obtained with THP-1 cells across different laboratories. In this study, we compared the effects of 5% O_2_, representing a physiologic normoxic level, and 18% O_2_, representing the atmospheric hyperoxic levels used in conventional tissue culture, on the proliferation, differentiation and primary macrophage functions of THP-1 cells grown with and without 2-ME and serum. Our studies indicate that altering the oxygen tension significantly influences THP-1 cell physiology, whereas omitting 2-ME and serum from the culture medium has minimal impact.

## Results

In all experiments, undifferentiated THP-1 cells were synchronized by serum starvation for 48 h prior to exposing cells to varying oxygen tension, 2-ME and serum. Synchronization aligns all cells at the same point in the cell cycle prior to initiating experimental manipulations. While synchronized cell populations are not common *in vivo,* synchronization of proliferating cell lines is a widely used experimental strategy to minimize variability in the experimental readout since cells in different stages of the cell cycle are well known to be differentially susceptible to and/or respond differently to environmental cues. Many experimental approaches have been described for synchronizing cells at specific phases of the cell cycle [15], and several common methods involve pharmacological agents acting at various points throughout the cell cycle [16], [17]. However, because of adverse cellular perturbations that can result from exposure to these pharmacological agents [18], we chose to use serum deprivation as the method for synchronizing undifferentiated THP-1 cells.

### Oxygen Tension does not Affect Proliferation of Undifferentiated THP-1 cells

**Figure 1:**
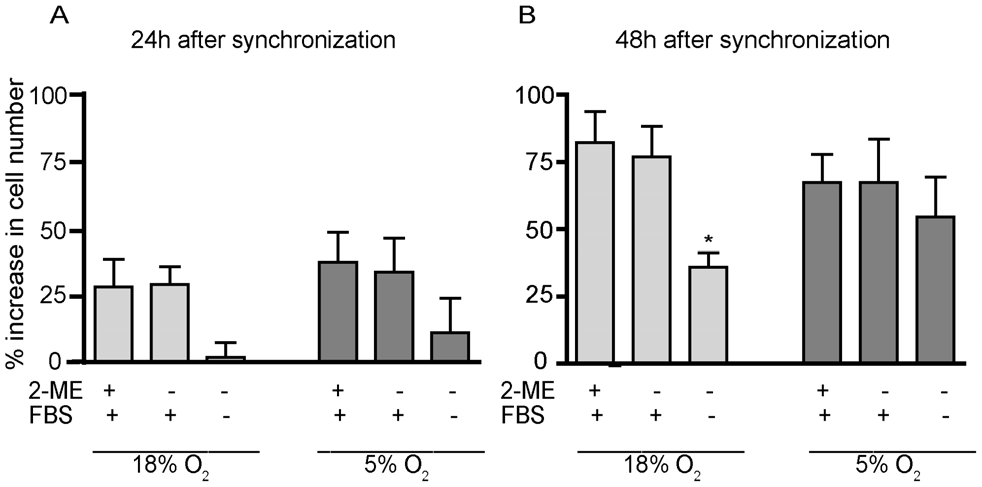
Influence of O_2_ tension, 2-ME and serum on proliferation of undifferentiated THP-1 cells. Monocytic THP-1 cells were synchronized by serum deprivation for 48 h and then cultured in hyperoxic (18% O_2_) or normoxic (5% O_2_) with or without 2-ME and/or FBS. Cell density was determined using a hemocytometer at 24 h (A) and 48 h (B) after synchronization. Data are presented as the mean ± SEM (n = 5 independent experiments). *Significantly different from cultures with 2-ME and FBS under the same oxygen tension at *p*<0.05 (one-way *ANOVA* with *post hoc* Tukey’s test).

A principal characteristic of undifferentiated THP-1 cells is their ability to proliferate in culture. Thus, we initially determined the influence of oxygen tension, 2-ME and serum on the proliferation of undifferentiated THP-1 cells. Specifically, we determined the percent increase in cell number at 24 and 48 h after synchronization in cultures maintained under 18% (hyperoxic) *versus* 5% (normoxic) O_2_ in the absence or presence of 2-ME and serum (Fig. 1). Across all treatment groups, the percent increase in cell number was greater at 48 h (Fig. 1B) relative to 24 h (Fig. 1A), suggesting that none of the treatments were overtly toxic to undifferentiated THP-1 cells. At either 24 or 48 h post-synchronization, there was no significant difference in the percent increase in cell number between cultures grown under 18% *versus* 5% O_2_. Removal of 2-ME from the culture medium had no effect on cell proliferation in cultures grown under either oxygen tension. In contrast, the removal of both 2-ME and serum significantly decreased cell proliferation in cultures grown under 18% O_2_, and a similar trend was observed in cultures grown under 5% O_2_, although the effect did not reach statistical significance.

### Oxygen Tension, 2-ME and Serum Influence the Metabolic Activity of THP-1 Cells

**Figure 2:**
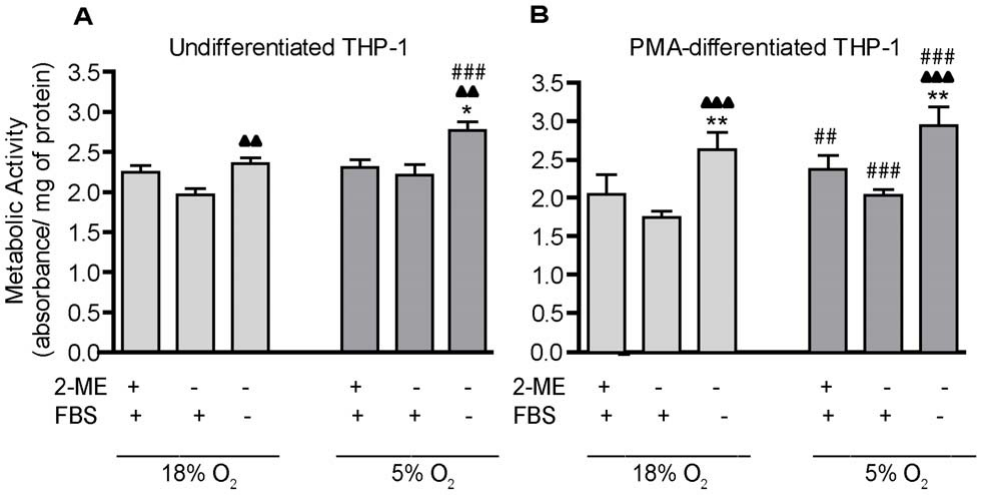
Influence of O_2_ tension, 2-ME and serum on the metabolic activity of THP-1 cells. THP-1 cells were synchronized by serum deprivation for 48 h. (A) Undifferentiated THP-1 cells were plated in 96-well plates precoated with poly-D-lysine. (B) THP-1 cells were triggered to undergo macrophage differentiation by incubating with PMA at 20 ng/ml for 24 h. MTT was added to both undifferentiated and differentiated THP-1 cells for 3 h under varying O_2_, 2-ME and serum conditions. MTT reduction, measured as the absorbance at 562 nm, was normalized to protein concentration. Data are presented as the mean ± SEM (n = 4 independent experiments. *Significantly different from +2-ME+FBS (standard culture conditions) under the same oxygen tension (one-way *ANOVA* and *post hoc* Tukey’s test); ^▴^Significantly different from –2-ME+FBS under the same oxygen tension (one-way *ANOVA* and *post hoc* Tukey’s test); ^#^Significantly different from the same culture condition in the 18% O_2_ group (e.g., 18% O_2_ *versus* 5% O_2_) by Student’s *t*-test. *, ^#^, ^▴^ *p*<0.05; **,^##^,^ ▴▴^ *p*<0.01; ***, ^###^, ^▴▴▴^ *p*<0.001.

While there was no evidence that removal of 2-ME and serum was overtly toxic to THP-1 cells, it is possible that the absence of these factors decreased cell viability resulting in decreased cell proliferation. To address this question, we next used the MTT assay to determine whether these culture conditions altered the metabolic activity of undifferentiated THP-1 cells. To account for differences in proliferation between cultures exposed to varying culture conditions, results from the MTT assay were normalized to protein concentrations in the same samples. Oxygen tension had no effect on metabolic activity in undifferentiated THP-1 cells cultured in the presence of both 2-ME and serum or with serum alone. Removal of both 2-ME and serum from the culture medium had no significant effect on metabolic activity in THP-1 cells cultured in 18% O_2_, but it significantly increased metabolic activity in cells cultured in 5% O_2_ (Figure 2A). These data suggest that the effect of serum on proliferation (Figure 1) is not due to effects on cell viability.

THP-1 cells can be stimulated to differentiate into macrophages by treatment with phorbol 12-myristate 13-acetate (PMA) [19], [20]. PMA induces cell cycle arrest followed by differentiation [21]. Quantification of metabolic activity in PMA-differentiated THP-1 cells indicated that similar to observations in undifferentiated THP-1 cells (Fig. 2A), culture in 5% O_2_ significantly increased metabolic activity (Fig. 2B). In differentiated THP-1 cells, however, this effect of oxygen tension was observed in the presence and absence of 2-ME and serum. Another difference between undifferentiated and PMA-differentiated THP-1 cells is that in the latter, removal of both 2-ME and serum significantly increased metabolic activity relative to cells cultured in the presence of both 2-ME and serum under either oxygen tension (Fig. 2B).

### Oxygen Tension, 2-ME and Serum Influence PMA-stimulated THP-1 Differentiation

**Figure 3:**
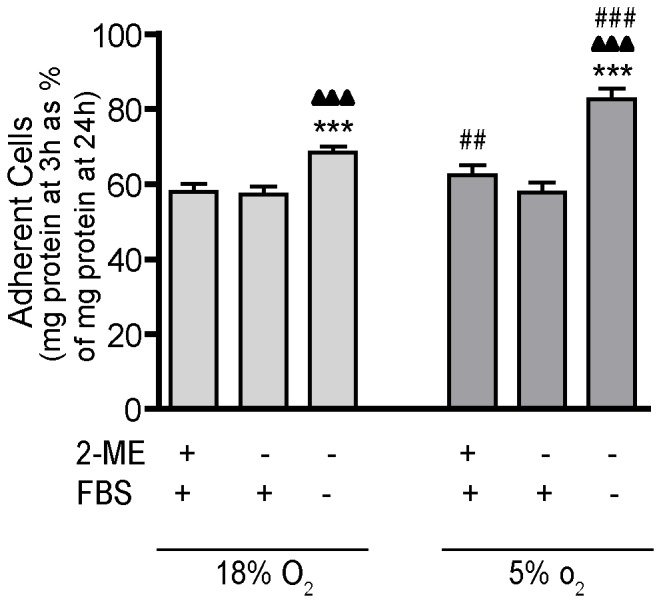
Influence of O_2_ tension, 2-ME and serum on the rate of THP-1 differentiation. Differentiation of THP-1 cells from monocytic to macrophage cells is associated with transition from a non-adherent to an adherent cell type. To determine whether culture conditions affect PMA-stimulated differentiation of THP-1 cells to macrophages, cell adhesion was assessed at 3 and 24 h after addition of PMA (20 ng/ml) to the culture medium. Data are presented as the mean ± SEM of the protein concentration of adherent cells at 3 h as a percentage of the protein concentration of adherent cells at 24 h (n = 4 independent experiments). *Significantly different from +2-ME+FBS (standard culture conditions) under the same oxygen tension (one-way *ANOVA* and *post hoc* Tukey’s test); ^▴^Significantly different from –2-ME+FBS under the same oxygen tension (one-way *ANOVA* and *post hoc* Tukey’s test); ^#^Significantly different from the same culture condition in the 18% O_2_ group (e.g., 18% O_2_ *versus* 5% O_2_) by Student’s *t*-test. **, ^##^,^ ▴▴^ *p*<0.01; ***, ^###^,^ ▴▴▴^ *p*<0.001.

Differentiation of THP-1 cells can be monitored phenotypically as a switch from a non-adherent to an adherent cell type. Thus, to evaluate the influence of culture conditions on THP-1 differentiation, we quantified cell adhesion at 3 h as a percentage of cell adhesion at 24 h after PMA stimulation in cultures maintained in 18% O_2_ *versus* 5% O_2_ in the presence or absence of 2-ME and serum. Relative to cultures exposed to 18% O_2_, differentiation was significantly accelerated at 3 h in cultures exposed to 5% O_2_ (Fig. 3). Under either oxygen tension, removal of 2-ME had no effect on cell adhesion at 3 h relative to cultures grown under standard culture conditions; however, removal of both 2-ME and serum significantly increased cell adhesion at 3 h (Fig. 3). Undifferentiated THP-1 cells that were not PMA-stimulated did not adhere when grown in serum free media for extended periods (data not shown).

### Oxygen Tension, 2-ME and Serum Influence β-hexosaminidase Release from Differentiated THP-1 Cells

**Figure 4:**
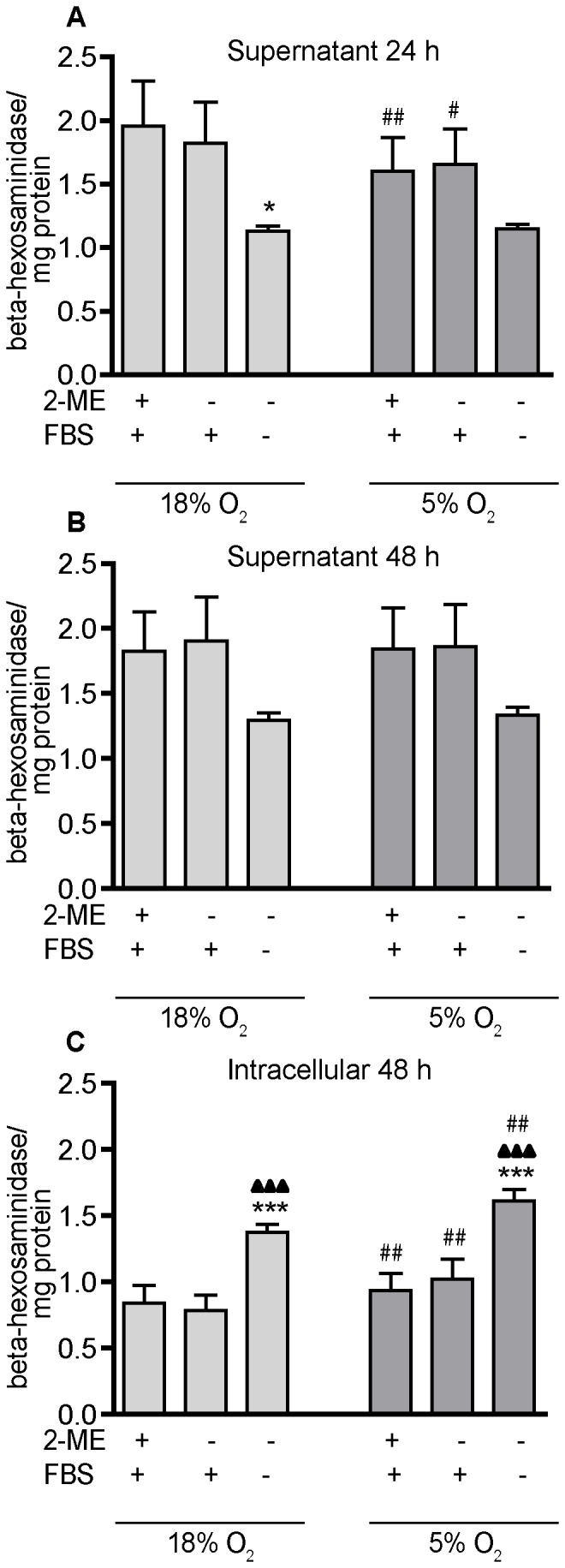
Influence of O_2_ tension, 2-ME and serum on release of β-hexosaminidase. Differentiated THP-1 cells constitutively release β-hexosaminidase that is measurable in the conditioned medium (supernatant) after 24 h (A) or 48 h (B) of culture. β-Hexosaminidase is also detected in cell lysates (C). β-Hexosaminidase activity per well was normalized to the concentration of protein in the same well as determined using the BCA protein assay. Data are presented as mean ± SEM (n = 3 independent experiments). *Significantly different from +2-ME+FBS (standard culture conditions) under the same oxygen tension (one-way *ANOVA* and *post hoc* Tukey’s test); ^▴^Significantly different from –2-ME+FBS under the same oxygen tension (one-way *ANOVA* and *post hoc* Tukey’s test); ^#^Significantly different from the same culture condition in the 18% O_2_ group (e.g., 18% O_2_ *versus* 5% O_2_) by Student’s *t*-test. *, ^#^, ^▴^ *p*<0.05; **, ^##^,^ ▴▴^ *p*<0.01; ***, ^###^,^ ▴▴▴^ *p*<0.001.

Critical to innate immune function is the constitutive release [22], [23] from macrophages of the lysosomal enzyme β-hexosaminidase [24]. To assess the effects of culture conditions on this macrophage function, we quantified both secreted and intracellular amounts of β-hexosaminidase in PMA-differentiated THP-1 cells cultured in 18% O_2_ *versus* 5% O_2_ in the absence or presence of 2-ME and serum. PMA-differentiated THP-1 cells released detectable quantities of β-hexosaminidase into the medium during 24 and 48 h of culture (Fig. 4A, B). This release was not dependent on stimulation by lipopolysaccharide (LPS) (data not shown), but it was influenced by oxygen tension, 2-ME and serum. The amount of β-hexosaminidase in the medium at 24 h was significantly decreased in cultures exposed to 5% O_2_ relative to 18% O_2_ in the presence of both 2-ME and serum or serum alone (Fig. 4A). However, by 48 h, this influence of oxygen tension on β-hexosaminidase release was no longer apparent (Fig. 4B). β-Hexosaminidase levels in the medium were also reduced by removal of both 2-ME and serum (Fig. 4A, B). While this effect was observed under both oxygen tension conditions and at both 24 and 48 h, it reached statistical significance only at the 24 h time point and only in cultures exposed to 18% O_2_.

To investigate whether the reduced β-hexosaminidase in the medium was due to decreased intracellular levels of β-hexosaminidase or decreased release from cells, we quantified intracellular levels of the enzyme, normalizing enzyme activity to total protein concentration. Culture in 5% O_2_ significantly increased intracellular β-hexosaminidase across all culture conditions, and removal of 2-ME and serum significantly increased intracellular levels of this enzyme in cultures exposed to either oxygen tension (Fig. 4C). These data suggest that reduced levels of β-hexosaminidase in the medium (Fig. 4A, B) reflect decreased release of this enzyme from PMA-differentiated THP-1 cells. While we did not measure the effects of oxygen tension on gene expression of β-hexosaminidase subunits, Cowan and collaborators [25] have previously shown that changing oxygen tensions did not alter mRNA levels of this enzyme in cardiomyocytes.

### Oxygen Tension Significantly Impacts the Phagocytic Activity of Differentiated THP-1 Cells

**Figure 5:**
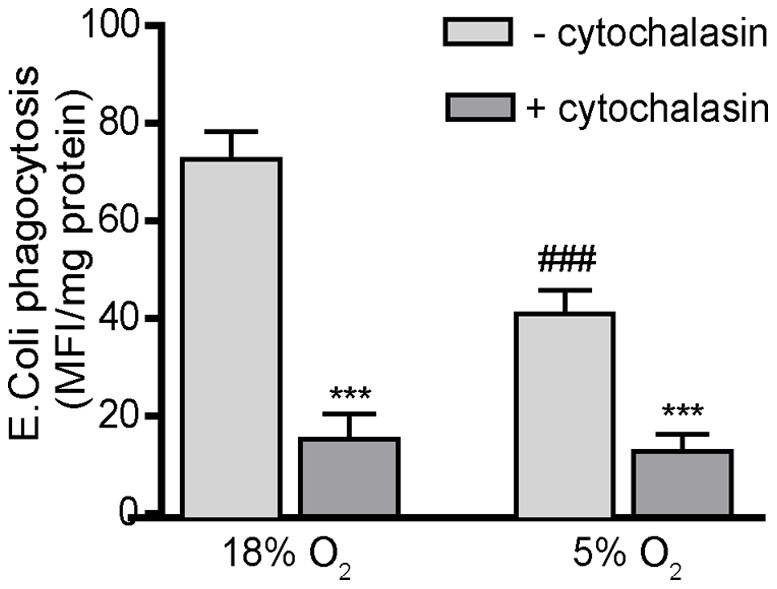
Oxygen tension significantly influences phagocytosis in PMA-differentiated THP-1 cells. Undifferentiated THP-1 cells were synchronized by serum deprivation for 48 h, plated at a density of 10^5^cells/well in a 96-well plate and differentiated with PMA (20 ng/ml) for 48 h in the absence of 2-ME and FBS. Differentiated THP-1 cells were washed and then incubated for 3 h with *E.coli* BioParticles®, which emit fluorescence upon acidification in lysosomes following phagocytosis. Phagocytosis, which was quantified by determining the fluorescence intensity at 600 nm, was blocked by pretreating cultures with cytochalasin D (2 µM) for 1 h prior to addition of *E. coli* BioParticles®. The mean fluorescence intensity was normalized to protein concentration as determined using the BCA protein assay. Data are presented as the mean ± SEM (n = 3 independent experiments). *Significantly different from control (– cytochalasin) treatment under the same oxygen tension; ^#^significantly different from the same culture condition in the 18% O_2_ treatment group (e.g., 18% O_2_ *versus* 5% O_2_) by Student’s *t*-test. ***, ^###^ *p*<0.001.

The essential role of macrophages in host-defense mechanisms is mediated in large part by their ability to phagocytose pathogens and cellular debris that contribute to inflammatory reactions and immune responses [26]. To quantify phagocytosis, we used pHrodo™ *E.coli* fluorescence conjugated BioParticles®. The fluorescence of these BioParticles® increases upon lysosomal uptake and subsequent acidification in the lysosomal compartment. Culturing PMA-differentiated THP-1 cells in 5% O_2_ significantly decreased phagocytosis of the *E. coli* BioParticles® relative to cells cultured in 18% O_2_ (Fig. 5). Pretreatment of cultures with cytochalasin-D decreased the mean fluorescence intensity by >75% in cultures exposed to *E. coli* BioParticles® under either oxygen tension, confirming that the fluorescence measured in these cultures was the result of phagocytosis of the BioParticles®.

To further evaluate the influence of oxygen tension on phagocytosis, THP-1 cells were PMA-differentiated at low or high oxygen levels for 24 h and switched to high or low oxygen, respectively, for 1 h immediately prior to adding the *E. coli* BioParticles®. Consistent with the data shown in Fig. 5, THP-1 cells differentiated in 5% O_2_ for 25 h phagocytize significantly fewer BioParticles® than cultures differentiated in 18% O_2_ for 25 h (Table 1). Differentiating THP-1 cells in 5% O_2_ for 24 h and then switching them to 18% O_2_ for 1 h significantly increased phagocytosis of the BioParticles® relative to all other treatment groups, including continuous 25 h exposure to 18% O_2_ (Table 1). Conversely, culturing the differentiating THP-1 cells in 18% O_2_ for 24 h with a subsequent 1 h incubation in 5% O_2_ significantly decreased BioParticle® uptake relative to continuous 25 h exposure to 18% O_2_, resulting in a level of phagocytosis that was comparable to that observed in cells cultured in 5% O_2_ continuously for 25 h (Table 1). These data suggest that phagocytosis is dependent on the oxygen tension during the phagocytosis assay and not on the oxygen tension during the PMA-induced differentiation, and that phagocytosis is increased at the higher (hyperoxic) oxygen tension, which is consistent with evidence that phagocytosis is dependent on the availability of extracellular oxygen for its respiratory burst [27], [28].

**Table 1:** Influence of oxygen tension on phagocytosis.

Oxygen Tension	*E.coli* phagocytosis
25 h @18% O_2_	67.15±2.23
25 h @ 5% O_2_	41.04±5.17 *
24 h @ 5% O_2_ → 1 h @ 18% O_2_	89.53×3.11 * ^ΔΔΔ^
24 h @ 18% O_2_ → 1 h @ 5% O_2_	46.07±5.56 *

THP-1 cells were cultured with PMA for 25 h to promote macrophage differentiation. In a subset of the cultures, the oxygen tension was switched from normoxic to hyperoxic or from hyperoxic to normoxic for the last hour of the incubation period. Phagocytosis was assessed as the uptake of *E.coli* BioParticles®. Data are presented as mean ± SEM (n = 3 per treatment group). *Significantly different from 25 h at 18% O_2_ at *p*<0.05; and ^ΔΔΔ^Significantly different from 25 h at 5% O_2_ and from 24 h at 18% O_2_ → 1 h @ 5% O_2_ at *p*<0.001 (one-way *ANOVA* with *post hoc* Tukey’s analysis).

### Oxygen Tension Influences NF-κB Activation and Cytokine and Chemokine Release in Differentiated THP-1 Cells

**Figure 6:**
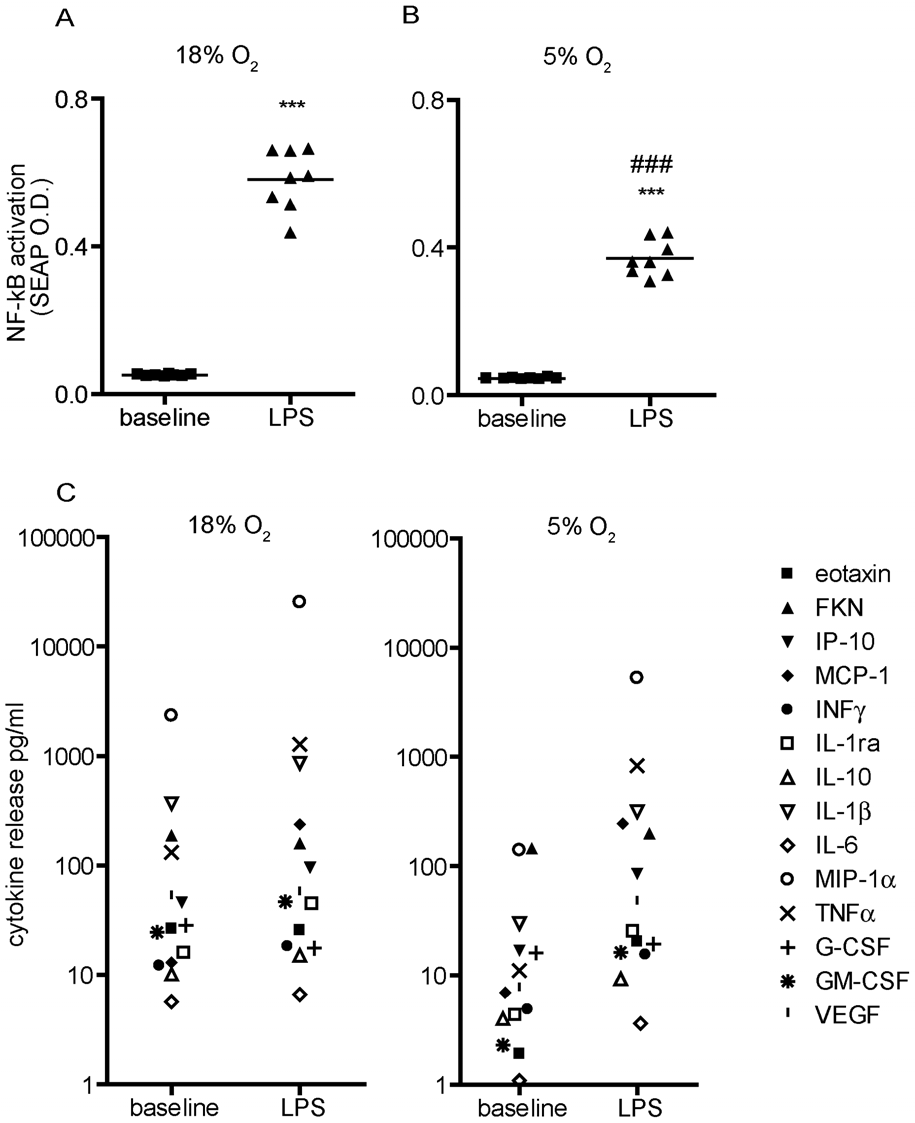
Oxygen tension influences LPS-induced NF-κB activation and release of cytokines in PMA-differentiated THP-1 cells. Undifferentiated THP-1 XBlue cells, which express an NF-κB reporter gene linked to secreted embryonic alkaline phosphatase (SEAP) were synchronized by serum deprivation for 48 h, and then differentiated with PMA (20 ng/ml) for 48 h in the absence of 2-ME and FBS. Differentiated THP-1 XBlue cells were then cultured in the absence (baseline) or presence of LPS (1 µg/ml) for an additional 24 h in either 18% (A, C) or 5% (B, D) O_2_. SEAP activity was quantified by QuantiBlue at 630 nm (A,B). Conditioned media from these cultures were analyzed using a human Milliplex Kit® to simultaneously quantify multiple cytokines and chemokines released from differentiated THP-1 cell during the 24 h incubation. Each symbol in panels C and D represents the mean of duplicates from one of five wells run in a representative experiment. Data are presented as the mean ± SEM (n = 2 independent experiments). ***Significantly different from baseline under the same oxygen tension at *p*<0.001,^ ###^significantly different from 18% O_2_ at *p*<0.001 (Student’s *t*-test).

A key intracellular signaling molecule that links various external stimuli to transcription of target genes in macrophages is NF-κB. NF-κB is a redox-responsive transcriptional factor, and its activation is a key regulator of the cellular response to oxidative stress [29]. NF-κB is also activated by LPS, which induces the expression of multiple genes encoding soluble mediators of inflammation, including cytokines, chemokines and growth factors [30], [31]. Thus, we next evaluated the effects of oxygen tension on NF-κB activation using THP-1 XBlue cells, which are stably transfected with an NF-κB-SEAP (secreted embryonic alkaline phosphatase) reporter gene. SEAP expression was measured in differentiated THP-1 XBlue cells cultured in 18% versus 5% O_2_ in the absence (baseline) or presence of LPS for 24 h. In the absence of LPS, oxygen tension had no effect on baseline levels of NF-κB activation (Fig. 6A, 6B). NF-κB was significantly activated by LPS relative to baseline levels under either oxygen tension; however, this response was attenuated in cells cultured in 5% O_2_ relative to cell cultured in 18% O_2_ (Fig. 6A, 6B).

A key question raised by these results is whether differential effects of oxygen tension on NF-κB activation translate into altered expression of cytokines and chemokines. To address this question, we used a multiplex cytokine array (specifically, the Milliplex Human Panel) to quantify 14 different cytokines and chemokines at the protein level in differentiated THP-1 cells cultured under different oxygen tensions in the absence or presence of LPS for 24 h. While no clear oxygen tension-related patterns emerged in the expression of individual cytokines or chemokines detected by the multiplex array either in the absence or presence of LPS (Fig. 6C and D), culturing differentiated THP-1 cells in 5% O_2_ caused a general decrease in baseline cytokine/chemokine expression levels and a greater upward shift from baseline with LPS stimulation (Fig. 6D).

**Figure 7:**
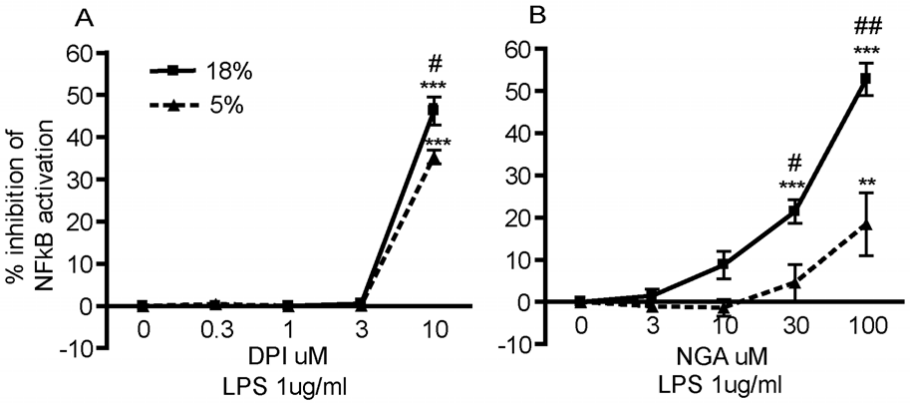
Oxygen tension influences redox in LPS-induced NF-κB activation in PMA-differentiated THP-1 cells. Undifferentiated THP-1 XBlue cells, which express an NF-κB reporter gene linked to secreted embryonic alkaline phosphatase (SEAP) were synchronized by serum deprivation for 48 h, and then differentiated with PMA (20 ng/ml) for 48 h. Differentiated THP-1 XBlue cells were then cultured in varying concentrations of DPI (A) or NGA (B) for 4 h followed by LPS (1 µg/ml) stimulation for an additional 24 h in either 18% or 5% O_2_. SEAP activity was quantified by QuantiBlue at 630 nm. Data are presented as the mean ± SEM (n = 2 independent experiments). *Significantly different from baseline (SEAP activity in the absence of inhibitor) at the same oxygen tension; **p<0.01; ***p<0.001 (one-way *ANOVA* with *post hoc* Tukey’s test). ^#^Significantly different from 5% O_2_ at same antioxidant concentration; ^#^ *p*<0.05; ^##^ *p*<0.01 (Student’s *t*-test).

A second key question raised by the differential effects of 18% versus 5% oxygen tension on LPS-induced NF-κB activation is whether this reflects differences in cellular ROS levels since NF-κB is a redox-responsive transcriptional factor [29]. To address this question, we determined whether by pretreating cells with inhibitors of ROS-generating sources (i.e., NADPH oxidase and lipoxygenase) would attenuate LPS-induced NF-κB activation and whether this attenuation would vary in magnitude between cultures grown under 18% versus 5% O_2_. Differentiated THP-1 cells were cultured under different oxygen tensions in the absence or presence of varying concentrations of the diphenylene iodinium (DPI), an NADPH oxidase inhibitor or nordihydroguaiaretic acid (NGA), an inhibitor of lipoxygenase, for 4 h followed by LPS stimulation for 24 h. Both DPI and NGA significantly inhibited LPS-induced NF-κB activation in a concentration-dependent manner in THP-1 cells grown under either oxygen tension, although a significantly greater inhibition was observed in cultures grown under 18% O_2_ relative to cultures grown under 5% O_2_ (Fig. 7A and B).

### Oxygen Uptake

Pericellular pO2, which is a primary determinant of oxygen-dependent cellular responses, is influenced by both atmospheric oxygen and the oxygen consumption of the cells. Thus, we used a Clark-type O_2_ electrode to determine whether mitochondrial oxygen consumption varied in differentiated THP-1 cells grown under 18% *versus* 5% O_2_ for 48 h after synchronization. The rate of mitochondrial oxygen consumption in cells grown under 5% O_2_ was 0.55 nmol O_2_×(min×0^6^ cells)^−1^; whereas cells grown under 18% O_2_ exhibited a slightly higher rate of oxygen consumption of 0.62 nmol O_2_×(min×10^6^ cells)^−1^. Under both oxygen tensions, rates of oxygen consumption were inhibited by more than 90% by addition of oligomycin (data not shown), indicating that most, if not all, oxygen uptake was linked to oxidative phosphorylation (i.e., mitochondrial ATP production). The ratio of the steady-state concentrations of oxygen around the cells grown at 18% vs. 5% was 3.8 as calculated using the cellular rates of oxygen uptake and the experimental concentration of oxygen in growth media at 20°C which is 262.2 µM. This ratio of 3.8 is similar to the ratio of the oxygen solubility at these two pO_2_, which is 4.2. Given that the rates of oxygen uptake of the cells grown at each pO_2_ were not dramatically different, the major determinant of the steady-state concentration of oxygen around the cells is the solubility of this gas at each of the pO_2_.

## Discussion

Our data demonstrate that adapting conventional culture conditions to more physiologically relevant conditions significantly alters THP-1 cell physiology. Specifically, we observed that while lowering oxygen tension from 18% O_2_ to 5% O_2_ had no effect on the proliferation of undifferentiated THP-1 cells, this endpoint was significantly altered by the removal of serum from the culture medium. Changing the oxygen tension from hyperoxic to normoxic did, however, significantly increase metabolic activity in both undifferentiated and differentiated THP-1 cells as well as enhance the differentiation of THP-1 cells and signficantly influence key aspects of macrophage function in differentiated THP-1 cells. Quantification of cellular uptake of oxygen in THP-1 cells grown under 18% O_2_ versus 5% O_2_ confirmed that the major determinant of the steady-state concentration of oxygen around these cells was the solubility of this gas at each pO_2_ and not cellular oxygen consumption. Removing 2-ME from the culture media had negligible effect on these endpoints. In contrast, removing both 2-ME and serum had significant effects on THP-1 metabolism, differentiation and macrophage functions under both conditions of oxygen tension, with more pronounced effects observed in THP-1 cells cultured under 5% O_2_.

Serum is commonly used as a supplement in cell culture to improve cell viability; however, there are a number of downsides including cost and the fact that serum is chemically undefined with high variability between batches. Adapting THP-1 cells to serum-free culture conditions would, therefore, significantly decrease costs and potentially increase culture consistency and experimental reproducibility. Removal of serum decreased proliferation of undifferentiated THP-1 cells; however, this effect was largely ameliorated by lowering the oxygen tension from 18% to 5% O_2_. This is consistent with previous studies demonstrating that the proliferation rate of peripheral blood mononuclear cells (PBMC) cultured in medium supplemented with a very low serum concentration was enhanced under normoxic conditions relative to hyperoxic conditions [32]. The decrease in cellular proliferation observed with the removal of serum (and 2-ME) was not due to decreased cell viability as evidenced by the fact that relative to their counterparts cultured in the presence of serum, THP-1 cells cultured in the absence of serum exhibited higher metabolic activity and faster differentiation. PMA-differentiated THP-1 cells cultured in the absence of serum also released approximately 30% less β-hexosaminidase, which correlated with significantly greater retention of β-hexosaminidase in the intracellular compartment. Thus, one of the most striking findings of our study was that the removal of serum from the culture medium for 48–96 hours enhanced THP-1 cell viability and function.

Hypoxic conditions have been shown to profoundly affect a broad range of myeloid cell properties *in vitro*, including expression of chemokine receptors and other cell-surface proteins, cytokine secretion, adhesion, migration, phagocytosis and cell survival [33], [34]. Thus, it is perhaps not surprising that lowering the oxygen tension from hyperoxic levels to normoxic levels altered macrophage functions in differentiated THP-1 cells. It has been reported that macrophages constitutively release small amounts of β-hexosaminidase independent of external stimuli [22], [23]. This continuous low-level release is important for maintaining the normal turnover of glycosaminoglycan in the tissue matrix; however, the release of hexosaminidases increases significantly during an inflammatory event and this contributes to the degradation of the surrounding tissue [35]. Differentiated THP-1 cells continuously release β-hexosaminidase under all the culture conditions tested, but the amount of enzyme released is significantly influenced by not only the removal of serum and 2-ME, as discussed above, but also by oxygen tension. Decreasing the oxygen tension to 5% O_2_ decreased β-hexosaminidase release coincident with increased intracellular levels of the enzyme. These data suggest that under normoxic conditions, the cells are better primed for responding to an inflammatory signal.

Phagocytosis by macrophages is an essential component of innate immunity. Previous studies have demonstrated that oxygen tension influences this activity. Pfau and collegues [36] demonstrated that peritoneal macrophages, which are exposed to normoxic conditions, and bone-derived macrophages cultured under low oxygen tension exhibed increased phagocytic activity relative to alveolar macrophages, which are exposed *in vivo* to hyperoxic conditions, and bone marrow-derived macrophages cultured under high oxgen tension. In contrast, there are numerous reports that alveolar macrophages have greater functional activity related to antimicrobial defense, including phagocytosis, compared to interstitial macrophages which display enhanced capabilities relevant to specific immune responses such as antigen processing as well as in antioxidant defenses [28], [37], [38]. Additional studies observed that hypoxia, *in vivo* and *in vitro*, increase the phagocytic activity of macrophages [39]. These apparent discrepancies may be attributed in part to the fact that phagocytosis is positively regulated by hypoxia-inducible factor-1α (HIF1α) [39], which is upregulated by hypoxia [40], and hypoxic conditions are typically associated with inflammatory processes [34]. In contrast, under normoxic conditions, HIF-1α is ubiquitinated and therefore less active [41]. Our data parallel these findings in that we observed that differentiated THP-1 cells exhibited increased phagocytosis when cultured under hyperoxic (18% O_2_) conditions relative to normoxic (5% O_2_) conditions. This result is also consistent with evidence that phagocytosis is dependent on the respiratory burst, which requires extracellular oxygen [27]. Collectively, these data implicate O_2_ or a metabolite in the acute regulation of phagocytosis. We tested this possibility by switching the oxygen tension under which differentiated THP-1 cells were maintained during the last hour of a 25 h incubation period. Regardless of the oxygen tension under which THP-1 cells were originally cultured, phagocytic activity was predominantly influenced by the oxygen tension during the specific time of phagocytosis, thus linking control of phagocytosis to environmental oxygen tension.

It is well documented that NF-κB activation requires an oxidative burst, which releases it from IκB [42]. Therefore, it was not particularly surprising that while LPS stimulated NF-κB in differentiated THP-1 cells grown under either oxygen condition, this response was attenuated in cells exposed to 5% O_2_ relative to 18% O_2_. NF-κB activation is closely linked to cytokine release in macrophages [43], so we predicted that LPS-stimulated release of cytokines would similarly be attenuated by culture in 5% O_2_. However, as indicated by multiplex cytokine analyses, differentiated THP-1 cells cultured in 5% O_2_ exhibited a more robust increase in cytokine release upon LPS stimulation relative to baseline than cells cultured in 18% O_2_. The concentrations of cytokines released upon LPS stimulation were comparable between cultures exposed to either oxygen tension, so the increased differential between baseline and LPS-induced levels of cytokine secretion in cells cultured under 5% O_2_ reflect the fact that the basal levels of cytokine secretion in the absence of LPS were significantly lower in these cultures relative to those exposed to 18% O_2_. Although the NF-κB activation baseline was not changed at 5% O_2_, these results from the cytokine release studies are consistent with results from the antioxidant studies showing enhanced inhibition of LPS-induced NF-κB activation at 18% O_2_ versus 5% O_2_, suggesting a more pronounced role for ROS signaling in THP-1 cells grown under 18% O_2_.

### Conclusions

In response to societal pressures to refine, reduce and replace the use of animals in experimentation, the increasing costs associated with animal models, and the advances in bioinformatics and systems biology, *in vitro* model systems are an increasingly important tool in biomedical science. While there are limitations associated with cell lines, particularly those that have been immortalized and thus express significant mutations that may alter the physiology of these cells relative to the primary cell type from which they were derived, cell lines, particularly those of human origin such as the THP-1 cell line, are especially useful for pilot projects, drug and toxicity screening, biochemical studies of signal transduction pathways and other types of studies that require large number of cells. Although widely used, standard tissue culture methods expose cells to oxygen levels considerably higher than those encountered by most cells under physiological conditions, and our data corroborate earlier studies in other cell types suggesting that altering oxygen tension impacts cell behavior. Regulating oxygen levels to optimize cell function *in vitro* is not unprecedented, and extensive and efficient use of this approach has been made in specific tissue culture models, including cultures of placental explant [44], embryos [45] and stem cells [2]. The present study confirms a major role for extracellular oxygen tension in modulating THP-1 cell physiology, which is consistent with literature documenting the regulation of immune function by the cellular redox environment both *in vivo* and in primary human monocytes and macrophages [46]. Our findings also suggest that THP-1 cells grown under tightly regulated oxygen tension in the absence of exogenous reducing agent are likely to provide a more physiologically relevant baseline from which to study the role of the local redox environment in regulating macrophage differentiation and function.

## Materials and Methods

### Cell Culture

Human monocytic THP-1 cells were purchased from American Type Culture Collection (ATCC) (Manassas, VA) [1]. THP1-XBlue cells, which were derived from the human monocytic THP-1 cell line, were purchased from Invitrogen (San Diego, CA). THP1-XBlue cells are NF-κB-reporter cells in which activation of the transcription factor NF-κB results in the secretion of embryonic alkaline phosphatase (SEAP), which is detected using Quanti-Blue reagent (Invitrogen). Cells were maintained in RPMI 1640 medium containing 11.11 mM glucose in but no phenol red (GIBCO, Carlsbad, CA) supplemented with 10% heat-inactivated fetal bovine serum (FBS, GIBCO), 1% penicillin (GIBCO), 1% streptomycin (GIBCO) and 50 µM 2-ME (Fisher Scientific, Pittsburgh, PA) at 37°C in a humidified incubator with 5% CO_2_ and 95% air (e.g., standard culture conditions). Prior to experimentation, the cells were starved for 48 h and subsequently cultured with or without 2-ME and/or FBS at 37°C in a humidified Thermo Scientific CO_2_ tissue culture incubator (NAPCO Series 8000WJ, Thermo Forma, Marietta, OH) equipped with built-in CO_2_ and O_2_ monitors and attached nitrogen and carbon dioxide gas supplies. Carbon dioxide was set to 5% v/v and oxygen to 5% of 18%. The oxygen and carbon dioxide contents of the incubator atmosphere were periodically verified using a Fyrite gas analyzer (Bacharach Inc., New Kensington, PA). For some experiments, cultures were treated for 24 or 48 h with phorbol 12-myristate 13-acetate (PMA, Sigma-Aldrich, St. Louis, MO) at 20 ng/ml to trigger THP-1 cells to undergo differentiation into macrophages [19], [20]. A stock solution of PMA at 40 µg/ml in dimethyl sulfoxide (DMSO, Sigma-Aldrich, Saint Louis, MO) was diluted in tissue culture medium with the final DMSO concentration of 0.1%. Addition of 0.1% DMSO alone did not cause THP-1 cells to undergo macrophage differentiation, nor did it affect their viability as assessed using the MTT assay (data not shown).

### Proliferation Assays

Non-differentiated THP-1 cells were synchronized by serum deprivation for 48 h prior to being plated at an initial density of 0.7×10^6^ cells/ml in 35 mm tissue culture dishes and cultured under the conditions indicated in Figure 1. At 24 or 48 h after plating, cell density was determined using a hemocytometer. The percent growth was calculated according the following equation: [(final cell density at 24 or 48 h *100)/(0.7×10^6^)] - 100). Experiments were independently repeated five times with 3 samples per treatment in each experiment.

### Metabolic Activity Assays

The metabolic activity of the cells was evaluated by quantifying the reduction of MTT (3-(4,5-dimethylthiazol-2-yl)-2,5-diphenyltetrazolium bromide, Sigma-Aldrich) to formazan, a reaction catalyzed by cellular reductases and dependent on the availability of reducing equivalents in the cell [47]. After synchronization (serum deprivation for 48 h), THP-1 cells were plated at 1×10^5^ cells/well in 96-well plates. To compare effects in monocytic THP-1 cells versus THP-1 macrophages, PMA (20 ng/ml) was added to the latter cultures 24 h prior to experimentation. For undifferentiated THP-1, cells were plated onto poly-D-lysine (100 µg/ml, Sigma-Aldrich)-coated wells immediately prior to initiating the experiment. MTT was added to the wells at 500 µg/ml final concentration and cells were incubated at 37°C under the culture conditions indicated in Figure 2 for 3 h. The supernatant was carefully aspirated and 5% triton X-100 (Fisher Scientific) in phosphate-buffered saline (PBS, 1 mM KH_2_PO_4_, 2.97 mM Na_2_HPO_4_^.^7H_2_O, and 155 mM NaCl, pH 7.4) was added to each well. The cells were then incubated at 37°C for 2 h prior to determining the optical density (OD) at 562 nm using a TECAN spectrophotometer (Spectra FLUOR Plus, Tecan Systems, Inc., San Jose, CA). Results were normalized against protein concentration as determined using the BCA™ Protein Assay Kit (Thermo Scientific, Rockford, IL, USA).

### Quantifying the Rate of THP-1 Macrophage Differentiation

Undifferentiated THP-1 cells were synchronized by serum deprivation for 48 h prior to re-plating at 1×10^5^ cells in 96-well plates in media containing 20 ng/ml PMA. Cells were then cultured at 37°C in 18% or 5% O_2_ with or without 2-ME and/or FBS. After 3 or 24 h of PMA stimulation, the non-adherent cells were removed with 3 rinses of PBS. The adherent cells were lysed with 50 µl of 1% triton X-100 in PBS, pH 7.4., and the protein content of the cell lysate was measured using the BCA protein assay. Cell adhesion was determined as the protein concentration of cultures at 3 h expressed as a percentage of the protein concentration at 24 h.

### Measurement of β-hexosaminidase

Spontaneous release of lysosomal contents of THP-1 macrophages was determined by measuring the enzyme β-hexosaminidase. Undifferentiated THP-1 cells were plated in 24-well plates at a density of 2×10^5^ cells/well and stimulated to differentiate by incubating with 20 ng/ml PMA for 24 or 48 h. After differentiation, conditioned medium was collected from each well and saved, and then cells were washed twice and lysed in 1% triton X-100 in PBS, pH 7.4. Triplicate aliquots of each conditioned medium and cell lysate sample (50 µl each) were mixed with an equal amount of substrate, 1.3 mg/ml *p*-nitrophenyl-*N*-acetyl-*β*-D-glucosaminide (Sigma-Aldrich), in 0.1 M citrate, pH 3.5. After incubation for 1 h at 37°C, 50 µl of 0.2 M glycine, pH 10.5, was added to stop the reaction, and the absorbance was measured at 405 nm using a TECAN spectrophotometer. Results were normalized against protein concentration in each sample, which was determined using the BCA protein assay. Experiments were independently repeated four times, and the results were comparable across all four experiments.

### Phagocytosis Assay

Phagocytosis was measured using the pHrodo™ *E.coli* fluorescence conjugated BioParticles® (Invitrogen/Molecular Probes, Eugene, OR). The fluorescence of the BioParticles® increases upon lysosomal uptake and subsequent acidification. Cells were synchronized for 48 h, plated at a density of 10^5^ cells/well in a 96-well plate and differentiated by PMA treatment for 48 h without 2-ME and FBS. Negative controls were incubated with 2 µM cytochalasin D (Sigma-Aldrich) for 1 h before the addition of the *E. coli* BioParticles® to inhibit phagocytosis. For some experiments, THP-1 were PMA-differentiated at low or high oxygen tension for 24 h and then switched to high and low oxygen tension, respectively, 1 h before the addition of the BioParticles®. Cells were incubated with BioParticles® for 90 minutes, washed and fluorescence was quantified using the Molecular Devices SpectraMax plate reader (Molecular Device, Sunnyvale, CA, USA) with the excitation wavelength set at 550 nm and the emission wavelength detection set at 600 nm. Results were normalized against protein concentration as determined using the BCA protein assay.

### NF-κB Activation

THP-1 XBlue cells grown without 2-ME and FBS were synchronized by serum deprivation for 48 h followed by PMA-differentiation for 48 h in 96-well plates at 1×10^5^ cells/well under 5% or 18% oxygen. Differentiated THP-1 XBlue cells were washed twice with PBS and stimulated by 1 µg/ml of lipopolysaccharide (LPS) derived from gram-negative bacteria (clone 055:B5, Sigma). NF-κB activation was determined by quantifying the secretion of embryonic alkaline phosphatase (SEAP), which was detected by Quanti-Blue reagent (Invitrogen) using a Synergy H1 microplate reader (BioTek Instruments, Inc., Winooski, VT). To determine whether oxidative stress influenced LPS-induced NF-κB activation, differentiated THP-1 XBlue cells were incubated for 4 h with diphenylene iodinium (DPI, Sigma) at 0.3–10 µM or nordihydroguaiaretic (NGA, Sigma) at 3–100 µM) before LPS stimulation.

### Quantification of Cytokine and Chemokine Release

THP-1 cells grown without 2-ME and FBS were synchronized by serum deprivation for 48 h followed by PMA-differentiation for 48 h under 5% or 18% oxygen. Differentiated THP-1 cells were plated at 0.5×10^6^ cell/ml in 6-well plates and cultured for an additional 24 h at 18% or 5% O_2_ in the absence (baseline) or presence of LPS at 20 ng/ml. Conditioned medium was collected from each well at the end of the 24 h incubation. A human Milliplex Kit (Millipore, Billerica, MA) was used to measure chemokine and cytokine concentrations in duplicate aliquots of each conditioned medium sample. This kit simultaneously interrogates 14 human cytokines, chemokines, and growth factors, including: IL-1β, IL-6, MIP-1α, IP-10, TNFα, IFNγ, IL-1ra, IL-10, INFγ, MCP-1, FKN, G-CSF, GM-CSF and VEGF. Samples were analyzed using the Bio-Plex array system, which includes a fluorescent reader and Bio-Plex Manager Analytic software (Bio-Rad, Hercules, CA). One hundred beads were counted for each analyte per well and cytokine concentrations (pg/ml) were calculated using Bio-Rad software.

### Oxygen Uptake

The oxygen uptake of intact THP-1 cell suspensions (6 to 7×10^6^ cells/ml) at 20°C was measured using a Clark-type O_2_ electrode from Hansatech (King’s Lynn, UK) [48]. Cells were incubated in the same RPMI 1640 culture medium used to maintain the cell line (e.g., medium containing 11.11 mM glucose but no phenol red). To evaluate mitochondria-derived oxygen uptake, measurements were repeated in the presence of 3 µM oligomycin (Sigma Chemical Company, Saint Louis, MO). A model for the steady-state concentration of oxygen was used that is based on the flow of oxygen delivered into the chamber and its *p*O_2_, the solubility of oxygen in the growth media (measured), and the oxygen uptake by cells in growth media (measured).

### Statistical Analysis

All data are presented as the mean ± SEM. Differences between 2 treatment groups were analyzed by Student’s *t*-test; whereas differences between >2 groups were determined by one-way *ANOVA* followed by Tukey’s post-test using GraphPad Prism 4 software (San Diego, CA). *p* values <0.05 were considered statistically significant.
